# Camrelizumab plus gemcitabine and oxaliplatin for the treatment of advanced intrahepatic cholangiocarcinoma: a bi-centric observational retrospective study

**DOI:** 10.3389/fonc.2023.1101038

**Published:** 2023-05-01

**Authors:** Yu-Qing Zhang, Kang Wang, Jin-Kai Feng, Lu-Yun Yuan, Chao Liang, Yan-Jun Xiang, Xu Wang, Fei-Fei Mao, Shu-Qun Cheng

**Affiliations:** ^1^ Cancer Center, Yueyang Hospital of Integrated Traditional Chinese and Western Medicine, Shanghai University of Traditional Chinese Medicine, Shanghai, China; ^2^ Department of Hepatic Surgery VI, Eastern Hepatobiliary Surgery Hospital, Second Military Medical University, Shanghai, China; ^3^ Department of Hepatobiliary Surgery, Yueyang Hospital of Integrated Traditional Chinese and Western Medicine, Shanghai University of Traditional Chinese Medicine, Shanghai, China; ^4^ Tongji University Cancer Center, Shanghai Tenth People’s Hospital, School of Medicine, Tongji University, Shanghai, China

**Keywords:** intrahepatic cholangiocarcinoma (ICC), immune checkpoint inhibitor (ICI), camrelizumab, gemcitabine and oxaliplatin (GEMOX), prognosis, tumor response, combination (combined) therapy

## Abstract

**Background:**

Immune checkpoint inhibitor (ICI), coupled with systemic chemotherapy, may enhance the clinical benefit of cancer by potentiating antitumor immunity, but its efficacy and safety are not clear in advanced intrahepatic cholangiocarcinoma (ICC). This study aims to assess the efficacy and safety of camrelizumab plus gemcitabine and oxaliplatin (GEMOX) for the treatment of advanced ICC in the real world.

**Methods:**

Advanced ICC patients receiving at least one session of camrelizumab plus GEMOX combination treatment from March 2020 to February 2022 at two high-volume centers were considered eligible. Tumor response was evaluated based on the Response Evaluation Criteria in Solid Tumors version 1.1 (RECIST v1.1). The primary endpoint was objective response rate (ORR), disease control rate (DCR), time to response (TTR), and duration of response (DOR). The secondary end points included overall survival (OS), progression-free survival (PFS), and treatment-related adverse events (TRAEs).

**Results:**

30 eligible ICC patients were enrolled and analyzed in this observational retrospective study. The median follow-up time was 24.0 (21.5–26.5) months. The ORR and DCR were 40% and 73.3%, respectively. The median TTR was 2.4 months and the median DOR was 5.0 months. The median PFS and OS were 7.5 months and 17.0 months, respectively. The most common TRAEs were fever (83.3%), fatigue (73.3%), and nausea (70%). Of all TRAEs, thrombocytopenia, and neutropenia were the most frequent severe AE (both 10%).

**Conclusion:**

The combination of camrelizumab and GEMOX is a potentially efficacious and safe treatment modality for advanced ICC patients. Potential biomarkers are needed to identify patients who might benefit from this treatment option.

## Introduction

Intrahepatic cholangiocarcinoma (ICC) is the second most common primary liver malignancy except for hepatocellular carcinoma in the world (1). The incidence of ICC varies from 1 to 5% of primary liver cancer in different surgical series, which has gradually increased in recent years (2–4). The etiology of ICC is mainly attributed to chronic hepatitis B and C virus infections. The proportion of cirrhosis in ICC patients is approximately 30% in Asian countries (2, 4, 5). About 30% of ICC patients are diagnosed at an advanced stage with synchronous metastases (3, 4, 6). For these patients (7), gemcitabine plus oxaliplatin (GEMOX) exhibits prolonged median overall survival (OS) and has become the first-line treatment option (8). Unsatisfactory, despite modern chemotherapeutic and surgical progress, the long-term prognosis has not achieved great improvement (9, 10). The 5-year OS rate of these patients is less than 10% using the standard treatment (11). Hence, it is clinically urgent to develop more effective treatment regime and improve the long-term outcome for patients with advanced ICC.

Immune checkpoint inhibitors (ICIs), such as anti-programmed cell death-1 (PD-1) antibodies, have demonstrated great clinical effects in various types of tumors (12). However, owing to the high heterogeneity and immunosuppression of tumor microenvironment (TME), the evidence to prove the therapeutic efficacy of GEMOX monotherapy in ICC patients is not sufficient yet (13). Recently, a single-arm phase II clinical trial demonstrated that the objective response rate (ORR) of 54%, and the median progression-free survival (PFS) and OS were 6.1 and 11.8 months respectively (14). Another two clinical studies using ICIs as a second-line treatment option in patients with ICC show promising results, too (15, 16). Interestingly, immunotherapy coupled with chemotherapy has also been investigated in many types of cancers and has shown inspiring anti-tumor efficacy (17, 18). The KEYNOTE 0189 and SHR-1210 study showed that OS in recurrent or metastatic non-small cell lung cancer (NSCLC) and nasopharyngeal carcinoma were significantly prolonged by a triple therapy consisting of pembrolizumab/camrelizumab combined with pemetrexed/gemcitabine and carboplatin/cisplatin (18, 19). However, whether this combination therapy is effective in advanced ICC has not yet been explored.

Camrelizumab is a novel humanized IgG4-κ PD-1 monoclonal antibody with high affinity combined with GEMOX regimen in advanced biliary tract cancer patients. Oxaliplatin in the GEMOX regimen had its immunological effects, which can potentiate the immune response of tumor cells, and synergize with PD-1/PD-L1 inhibitors, such as camrelizumab. Several phase II clinical trials have reported that camrelizumab can achieve good tumor control in biliary tract cancer including ICC (14, 20). Herein, in this bi-centric observational retrospective study, we focused on a real-world cohort of advanced ICC patients to assess the safety and anti-tumor activity of the combined therapy comprising of camrelizumab plus GEMOX.

## Methods

### Study design and patients

This observational retrospective study was conducted in consecutive advanced ICC patients who received camrelizumab plus GEMOX treatment in the two participating hospitals, the Yueyang Hospital of Integrated Traditional Chinese and Western Medicine and the Eastern Hepatobiliary Surgery Hospital from March 2020 to February 2022. The study protocol and amendments were reviewed and approved by the Institutional Ethics Committees of each center. The study was conducted in compliance with the Good Clinical Practice guidelines, and the ethical principles of the Declaration of Helsinki. The requirement for written informed consent was waived due to the retrospective nature of this study. Patients’ personal identities were kept anonymized to protect their secrecy.

### Eligibility criteria

The inclusion criteria were (I) treatment-naive advanced ICC diagnosed by histopathology, computed tomography (CT) or magnetic resonance imaging (MRI); (II) camrelizumab combined with GEMOX as first-line systemic therapy; (III) Child-Pugh class A or selected B liver function (scores ≤7); (IV) an Eastern Cooperative Oncology Group (ECOG) performance status score of 0–1; (V) presence of at least one measurable lesion assessed using the Response Evaluation Criteria in Solid Tumors version 1.1 (RECIST v1.1); and (VI) had sufficient organ function and an estimated life expectancy more than 3 months. The exclusion criteria were patients with (I) previous locoregional or systemic therapy; (II) other combined treatment besides camrelizumab and GEMOX; (III) a history of other malignancies; and (IV) incomplete clinical data or lost to follow-up.

### Treatment protocols

Enrolled patients received intravenous administration of camrelizumab at a standard dose of 3mg/kg every 3 weeks (total dose ≤ 200mg) plus gemcitabine of 1000 mg/m^2^ every 2 weeks, and oxaliplatin of 85–100 mg/m^2^ once a week, with possible dose adjustments according to the drug’s instructions. GEMOX chemotherapy and camrelizumab immunotherapy lasted for no more than 12 cycles. Once chemotherapy intolerance, immune-related severe toxicity, or disease progression occurred, the triple treatment regimen was discontinued. Other second-line treatment option for advanced ICC, such as 5-fluorouracil combined with oxaliplatin (FOLFOX) was recommended.

### Assessment and follow-up

The pre-treatment baseline laboratory indicators, such as blood routine test and biochemistry, tumor biomarker test, and blood coagulation test, were obtained before the initiation of the first cycle of camrelizumab and GEMOX therapy. After the first cycle of the triple combination therapy, tumor response was assessed every 4–8 weeks using imaging examinations which included contrast-enhanced computed tomography (CT) or magnetic resonance imaging (MRI) based on RECIST (version 1.1) criteria. Treatment-related adverse events (TRAEs) and laboratory abnormalities were tightly monitored and recorded from the time of treatment onset till 90 days after cessation of treatment, and were graded on the basis of the National Cancer Institute Common Terminology Criteria for Adverse Events (version 5.0). If multiple instances of the same type of toxicity occurred, the highest grade for each patient in a given category was adopted.

All patients were regularly followed up by the attending physicians every 1–2 months. At each follow-up visit, routine physical examinations, laboratory blood tests, and imaging examinations were performed to monitor disease status and adverse effects. This study was censored on March 31^st^ 2022.

### Study outcomes

The primary endpoints were objective response rate (ORR), disease control rate (DCR), time to response (TTR), and duration of response (DOR). ORR was defined as the proportion of patients with complete response (CR) and partial response (PR). DCR was defined as the proportion of patients with CR, PR, and stable disease (SD). TTR was defined as time from the first drug administration to the first tumor response. DOR was defined as the interval from the first tumor response to radiologically confirmed disease progression. The secondary endpoints were overall survival (OS), progression-free survival (PFS), and TRAEs. OS was defined as time from the first drug administration to death from any cause. PFS was defined as the time from the first drug administration to the first documented disease progression or death from any cause.

### Statistical analysis

Survival curves of OS and PFS were generated using the Kaplan-Meier method. Continuous variables were reported as median (interquartile range) or mean ± standard deviation (SD) according to the distribution of data (normal or skewed). Categorical variables were described as numbers and percentages. Univariate Cox regression analysis was performed to identify the potential variables which were closely associated with patients’ prognosis. Variables which were significantly related to patients’ prognosis (*P* < 0.05) were incorporated into multivariate Cox regression analysis to identify independent risk factors of OS and PFS. All statistical analyses were performed using GraphPad Prism, Version 8.2.0 (GraphPad, Inc.) and SPSS 26.0 software (SPSS Inc., Chicago, IL, USA). Statistical significance was set at *P* value less than 0.05.

## Results

### Baseline demographic and clinical characteristics of patients

**Figure 1:**
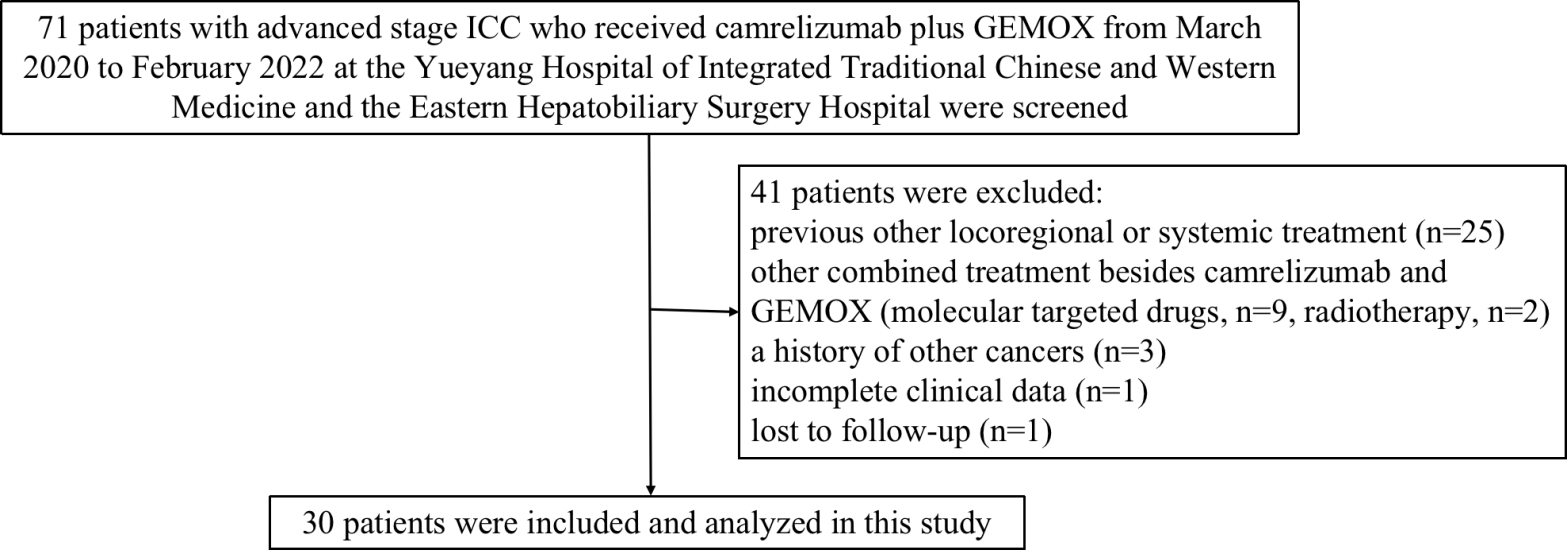
Study design and flow diagram of this study. ICC, intrahepatic cholangiocarcinoma; GEMOX, gemcitabine and oxaliplatin.

Seventy-one patients with advanced stage ICC who received camrelizumab combined with GEMOX were screened for eligibility from the electronic medical system of the two participating hospitals. Of these patients, 41 patients were considered ineligible because they did not meet the inclusion criteria or met the exclusion criteria (**Figure 1**). Finally, a total of 30 patients treated with at least one session of camrelizumab coupled with GEMOX were included and analyzed in this study.

The median follow-up time of the cohort of patients was 24.0 months (range

21.5–26.5 months). The reason of treatment discontinuation was progressive disease in 8 (26.7%) patients, and severe adverse events in 3 (10%) patients. The baseline demographic and clinical characteristics of advanced ICC patients are listed in **Table 1**. The median age of patients was 64 years old, and two-thirds of patients were males. Twenty-one (70.0%) patients had ECOG performance status score of 1. Twenty-four (80.0%) patients had Child-Pugh class A liver function, and the remaining 6 (20.0%) patients had Child-Pugh class B liver function. Seven (23.3%) patients had hepatitis B virus (HBV) infection background, and 23 (76.7%) patients had non-HBV-related ICC. Four (13.3%) patients had biliary obstruction before chemotherapy, which was successfully treated by biliary drainage. Six (20.0%) patients were associated with cirrhosis. Twenty-one (70.0%) patients had tumor size over 5 cm. Five (16.7%) patients had serum alpha-fetoprotein (AFP) level ≥ 20 ng/ml, 23 (76.7%) patients had protein induced by vitamin K absence or antagonist-II (PIVKAII) ≥ 20 mAU/ml, and 18 (60%) patients with a CA-199 level ≥ 40 U/ml. 18 (60%) patients had concurrent metastasis, of whom 16 had lung metastasis and 2 had bone metastasis. The median cycles of triple combination therapy were 7 (range, 2–10).

**Table 1:** Baseline demographic and clinical characteristics of advanced intrahepatic cholangiocarcinoma patients.

Characteristics	All patients (n=30)
Age, median (range), years	64 (44–75)
< 60	13 (43.3%)
≥ 60	17 (56.7%)
Gender	
Male	20 (66.7%)
Female	10 (33.3%)
ECOG performance status	
0	9 (30.0%)
1	21 (70.0%)
Child-Pugh class	
A	24 (80.0%)
B	6 (20.0%)
Etiology	
HBV	7 (23.3%)
Non-HBV	23 (76.7%)
Cirrhosis	
Yes	6 (20.0%)
No	24 (80.0%)
PT (s), median (Q1, Q3)	12.1 (11.0–12.9)
INR, median (Q1, Q3)	1.01 (0.91–1.08)
WBC (*10^9/L), mean ± SD	7.6 ± 3.3
RBC (*10^12/L), mean ± SD	3.9 ± 0.8
PLT (g/L), mean ± SD	221 ± 95
TBil (μmol/L), median (Q1, Q3)	14.9 (9.8–32.2)
ALB (g/L), mean ± SD	37.6 ± 7.7
ALT (U/L), median (Q1, Q3)	51 (24.5–81.5)
AST (U/L), median (Q1, Q3)	42.5 (23–68)
GGT (U/L), median (Q1, Q3)	213.5 (70.5–338.8)
ALP (U/L), median (Q1, Q3)	183 (95.3–417)
BUN (mmol/L), median (Q1, Q3)	4.46 (3.33–5.49)
Creatinine (μmol/L), median (Q1, Q3)	69 (54–79)
Glucose (mmol/L), median (Q1, Q3)	5.16 (4.67–5.49)
AFP (ng/mL), median (Q1, Q3)	3.5 (2.4–9.3)
< 20	25 (83.3%)
≥ 20	5 (16.7%)
PIVKAII (mAU/mL), median (Q1, Q3)	24 (19.75–29.75)
< 20	7 (23.3%)
≥ 20	23 (76.7%)
CA-199 (U/mL)	
< 40	12 (40.0%)
≥ 40	18 (60.0%)
Tumor size (cm)	
< 5	9 (30.0%)
≥ 5	21 (70.0%)
Extrahepatic metastases	
Yes	18 (60.0%)
No	12 (40.0%)
Metastatic sites	
Lung	16 (88.9%)
Bone	2 (11.1%)
Cycles of treatment, median (range)	7 (2–10)

ECOG, Eastern Cooperative Oncology Group; HBV, hepatitis B virus; PT, prothrombin time; INR, international normalized ratio; WBC, white blood cell; RBC, red blood cell; PLT, platelet; TBil, total bilirubin; ALB, albumin; ALT, alanine aminotransferase; AST, aspartate aminotransferase; GGT, γ-glutamyltranspeptidase; ALP, alkaline phosphatase; BUN, blood urea nitrogen; AFP, alpha-fetoprotein; PIVKAII, protein induced by vitamin K absence or antagonist-II; CA-199, carbohydrate antigen-199; Q1, lower quartile; Q3, upper quartile.

### Efficacy

As of the data cut-off on March 31st, 2022, 18 (60%) patients died or had disease progression. Based on the RECIST v1.1 criteria, 5 (16.7%) patients achieved CR, 7 (23.3%) patients achieved PR, SD was observed in 10 (33.3%) patients, and PD was reported in 8 (26.7%) patients. Therefore, in this study, the ORR and DCR of the triple therapy were 40% and 73.3%, respectively (**Table 2**). The median time to response was 2.4 months (range 1.5–12.6). The median duration of response was 5.0 months (range 2.4–18.5) months (**Table 2**).

**Table 2:** Clinical efficacy of camrelizumab plus GEMOX in patients with advanced intrahepatic cholangiocarcinoma.

Variables	Camrelizumab + GEMOX (n=30)
Objective response	
No. of response	12
% of patients	40.0
Disease control	
No. of disease control	22
% of patients	73.3
Best overall response–no. (%)	
Complete response	5 (16.7)
Partial response	7 (23.3)
Stable disease	10 (33.3)
Progression disease	8 (26.7)
Time to response, months	
Median	2.4
Range	1.5–12.6
Duration of response, months	
Median	5.0
Range	2.4–18.5

**Figure 2:**
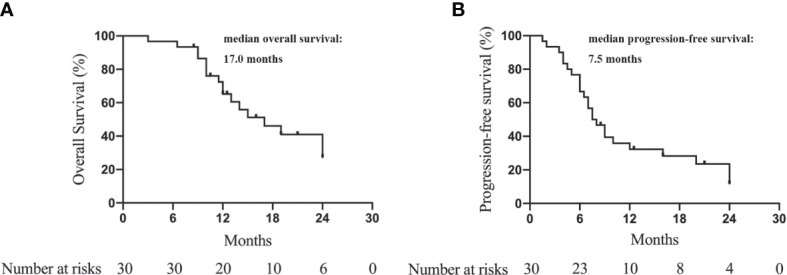
Kaplan-Meier curves to show the overall survival **(A)** and progression-free survival **(B)** of advanced ICC patients treated with camrelizumab and GEMOX.

The median OS was 17.0 months (95% CI = 10.3–23.7 months). The 6-, 12-, 18-, and 24-month OS was 94.3%, 65.2%, 43.5%, and 27.3%, respectively. (**Figure 2A**). The median PFS was 7.5 months (95% CI = 5.4–9.6 months). The 6-, 12-, 18-, and 24-month PFS were 66.7%, 32.3%, 25.3%, and 11.8%, respectively (**Figure 2B**).

### Independent prognostic factors

Univariate and multivariate analysis showed that tumor size ≥5 cm (*P* < 0.001), ALB <35 g/L (*P* < 0.001), PIVKAII ≥20 mAU/mL (*P* = 0.001), cirrhosis (*P* < 0.001), and tumor RECIST response (*P* < 0.001) were independent risk factors of OS (**Table 3**). Tumor size ≥5 cm (*P* < 0.001), AFP ≥20 ng/L (*P* < 0.001), ALT ≥40 U/L (*P* = 0.006), cirrhosis (*P* = 0.002), and tumor RECIST response (*P* = 0.008) were independent risk factors of PFS (**Table 4**).

**Table 3:** Univariate and multivariate Cox regression analysis to identify independent prognostic factors of OS in advanced ICC patients treated with Camrelizumab and GEMOX.

Clinical Factors	Univariate analysis HR (95% CI)	*P*	Multivariate analysis HR (95% CI)	*P*
Tumor size (≥5 vs. <5 cm)	1.88 (1.54–2.30)	**< 0.001**	1.73 (1.40–2.13)	**< 0.001**
ALB (≥35 vs. <35 g/L)	0.52 (0.40–0.67)	**< 0.001**	0.62 (0.48–0.81)	**< 0.001**
TBil (≥17.1 vs. <17.1 μmol/L)	1.05 (0.85–1.29)	0.652		
AFP (≥20 vs. <20 ng/L)	1.11 (0.86–1.44)	0.415		
PIVKAII (≥20 vs. <20 mAU/mL)	1.73 (1.42–2.12)	**< 0.001**	1.43 (1.16–1.76)	**0.001**
ALT (≥40 vs. <40 U/L)	1.20 (0.98–1.46)	0.077		
Liver cirrhosis (Yes vs. No)	1.54 (1.21–1.96)	**< 0.001**	1.62 (1.27–2.07)	**< 0.001**
Tumor response (Yes vs. No)	0.60 (0.48–0.73)	**< 0.001**	0.64 (0.52–0.78)	**< 0.001**

Bold values indicate statistical significance (P < 0.05).

ALB, albumin; TBil, total bilirubin; AFP, alpha-fetoprotein; PIVKAII, protein induced by vitamin K absence or antagonist-II; ALT, alanine aminotransferase.

**Table 4:** Univariate and multivariate Cox regression analysis to identify independent prognostic factors of PFS in advanced ICC patients treated with Camrelizumab and GEMOX.

Clinical Factors	Univariate analysis HR (95% CI)	*P*	Multivariate analysis HR (95% CI)	*P*
Tumor size (≥5 vs. <5 cm)	1.84 (1.54–2.20)	**< 0.001**	1.72 (1.42–2.08)	**< 0.001**
ALB (≥35 vs. <35 g/L)	0.61 (0.48–0.79)	**< 0.001**	0.80 (0.61–1.04)	0.096
TBil (≥17.1 vs. <17.1 μmol/L)	1.14 (0.95–1.37)	0.173		
AFP (≥20 vs. <20 ng/L)	1.51 (1.22–1.88)	**< 0.001**	1.47 (1.19–1.83)	**< 0.001**
PIVKAII (≥20 vs. <20 mAU/mL)	1.40 (1.17–1.68)	**< 0.001**	1.19 (0.98–1.43)	0.073
ALT (≥40 vs. <40 U/L)	1.41 (1.18–1.69)	**< 0.001**	1.30 (1.08–1.56)	**0.006**
Liver cirrhosis (Yes vs. No)	1.37 (1.12–1.69)	**0.003**	1.40 (1.14–1.73)	**0.002**
Tumor response (Yes vs. No)	0.77 (0.64–0.92)	**0.005**	0.78 (0.65–0.94)	**0.008**

Bold values indicate statistical significance (P < 0.05).

ALB, albumin; TBil, total bilirubin; AFP, alpha-fetoprotein; PIVKAII, protein induced by vitamin K absence or antagonist-II; ALT, alanine aminotransferase.

### Safety

Treatment-related adverse events (TRAEs) including frequency and severity grade were evaluated according to CTCAE, version 5.0. At least one AE occurred in 25 (83.3%) patients (**Table 5**). The most common TRAEs were fever (83.3%), fatigue (73.3%), nausea (70%), and thrombocytopenia (66.7%). The grade 3 or worse TRAEs recorded in our study were thrombocytopenia(10%), neutropenia (10%), and hypokalemia (6.7%).

**Table 5:** Summary of the treatment-related adverse events.

AE term, n(%)	Any grade	Grade 3/4
Fever	25 (83.3%)	0
Fatigue	22 (73.3%)	0
Nausea	21 (70%)	0
Thrombocytopenia	20 (66.7%)	3 (10%)
Hypocalcemia	19 (63.3%)	0
Decreased appetite	19 (63.3%)	0
Hypokalemia	18 (60%)	2 (6.7%)
Neutropenia	17 (56.7%)	3 (10%)
AST increased	17 (56.7%)	0
Fatigue	16 (53.3%)	0
ALT increased	15 (50%)	0
Anemia	10 (33.3%)	0
Vomiting	10 (33.3%)	0
Diarrhea	7 (23.3%)	0
Serum bilirubin increase	6 (20%)	0
Pruritus	5 (16.7%)	0
Insomnia	5 (16.7%)	0
Skin pigmentation	4 (13.3%)	0
Albumin decreased	3 (10%)	0
Hypomagnesemia	2 (6.7%)	0
Hypophosphatemia	2 (6.7%)	0
Creatinine increased	2 (6.7%)	0
Proteinuria	1 (3.3%)	0
Alopecia	1 (3.3%)	0
Arthralgia	1 (3.3%)	0

AE, adverse event; AST, aspartate transaminase; ALT, alanine aminotransferase.

## Discussion

Present treatment options for advanced ICC are limited. Systemic chemotherapy is considered to be the standard treatment for advanced ICC. However, the tumor response rate of advanced ICC patients to chemotherapy alone is not very satisfactory. TOPAZ-1 trial recommended durvalumab (a PD-L1 antibody) plus gemcitabine and cisplatin (GemCis) as one of the first-line regimens for unresectable and metastatic BTC. Nevertheless, the ICI used in this study targets PD-L1, which delivers various safety and efficacy profile from anti-PD-1. Whether PD-1 inhibitors can augment the therapeutic efficacy of GEMOX chemotherapy is unclear.

To the best of our knowledge, this is the first study regarding systemic treatment consisting of anti-PD-1 antibody plus chemotherapy in patients with advanced ICC and investigating the efficacy and safety of the combined therapy. In this study, PD-1 plus GEMOX showed a promising anti-tumor activity in patients with advanced ICC. Tumor response of ORR and DCR reached 40% and 73.3%, respectively, on the basis of RECIST v1.1. The ORR of camrelizumab plus GEMOX was much higher than immunotherapy or chemotherapy alone. OS and PFS of these patients achieved 17.0 and 7.5 months, respectively.

Many retrospective studies reported long-term outcomes after surgical resection of ICC, and the median OS after radical surgery ranges from 4 to 48 months (5). The ABC-02 trial demonstrated that the median PFS and OS of advanced ICC patients treated with GEMOX were 8.0 and 11.7 months, respectively (21). The PFS of our cohort was slightly shorter than that of the ABC-02 trial. First, the ABC-02 trial investigated and compared the survival outcomes of patients with locally advanced or metastatic biliary cancer, so the study population was different. Second, the GP regimen (gemcitabine plus cisplatin) was used in the ABC-02 trial, so chemotherapeutic approaches were various.

A phase II study reported that GEMOX chemotherapy regimen obtained an ORR of 14.9% –18.9% as first-line treatment of advanced ICC (22), and the Korean Cancer Group yielded an ORR of 18.9% (23). In a retrospective study that investigated TACE in 50 ICC patients, the median OS was 12.3 months with an ORR of 70% (24). Another multicenter retrospective study observed the treatment efficacy of gemcitabine plus platinum-based chemotherapy for 30 advanced ICC patients. The PFS was 7.0 months and OS was 14.2 months (25). Our study confirmed that a combination therapy of camrelizumab plus GEMOX could provide a better prognosis and tumor response compared with these historical trials. This combination treatment improved the overall response rate by more than 20%. The median OS and median PFS were also prolonged by camrelizumab plus GEMOX compared with GEMOX alone (16.0 months vs. 8.8 months; 7.0 months vs. 3.4 months, respectively) (22).

Many trials also used immunotherapy as second-line therapy. Patients with advanced ICC who had initially received 1–3 lines of nivolumab treatment achieved an ORR of 22% (21). Among patients with locally advanced ICC, disease control was observed in about 60% of patients, as reported by Valle and his colleagues (21). The combination of gemcitabine, oxaliplatin, and bevacizumab has also been investigated in advanced biliary-tract cancers with median PFS of 7 months and median OS of 21.7 months (26).

However, the majority of advanced ICC patients had either immune-excluded or immune-desert phenotypes. These patients had a poor response to PD-1/PD-L1 inhibitors monotherapy (27). Several ongoing trials are investigated the safety and efficacy of the combination of immunotherapy and chemotherapy in advanced ICC, such as pembrolizumab plus gemcitabine/cisplatin (NCT04003636), pembrolizumab plus capecitabine/oxaliplatin (NCT03111732), and nivolumab plus gemcitabine and cisplatin (NCT03101566). Significantly, the immunosuppressive effects of VEGF, such as upregulation of immune checkpoints, decrease of CD8^+^ T-cell infiltration and function, and increase of immunosuppressive cell subtypes, could be dramatically reversed by bevacizumab and chemotherapy (28, 29). On account of these clinical evidence and fundamental mechanisms, combination of systemic chemotherapy and immunotherapy has been widely applied in clinical practice for the treatment of advanced ICC.

Furthermore, the safety of the novel systemic combination treatment is acceptable (30). Of note, thrombocytopenia (10%) and neutropenia (10%) were the most frequent grade 3–4 TRAEs associated with this combination therapy, followed by hypokalemia (6.7%). The incidence of severe AEs was notably lower than that of the trial using combination of camrelizumab and GEMOX in advanced BTC (14) and another study (48%) reported by Fang et al. (31), implying that the united regime was a safe treatment option. The common grade 1–2 toxicities associated with the use of camrelizumab and GEMOX, such as fever and fatigue, were manageable and reversible with dose adjustment and appropriate supportive care. In clinical practice, the treatment benefits and risks of chemotherapeutic and immune-related toxicity following this combination therapy should be carefully evaluated.

The present study had some limitations. First, it is a retrospective study, leading to potential selection bias and insufficient medical evidence. Second, this study is single-arm with no control group, so it is impossible to compare the efficacy and safety of this combined therapy with the standard therapeutic approaches. Third, the substantial heterogeneity of the study population and the inconformity of treatment regimens may influence the interpretation of our findings. Thus, the efficacy and safety of camrelizumab plus GEMOX need to be further explored in a randomized controlled study with a larger sample size. Last, It was reported that the expression level of PD-1/PD-L1 on tumor cells or tumor-infiltrating immune cells was associated with treatment response (32). Nevertheless, the clinical usefulness of these potential biomarkers was not investigated in this study.

## Conclusion

To sum up, camrelizumab plus GEMOX has promising efficacy and provides acceptable safety in patients with advanced ICC. Our findings provide a potential treatment option and a certain basis for further study of this regimen in advanced ICC. Furthermore, to select patients who are most likely to benefit from the combination therapy, the identification of molecular biomarkers is needed in the future.

## Data availability statement

The raw data supporting the conclusions of this article will be made available by the authors, without undue reservation.

## Ethics statement

This study was conducted in accordance with the Declaration of Helsinki and approved by the Yueyang Hospital of Integrated Traditional Chinese and Western Medicine and Eastern Hepatobiliary Surgery Hospital (EHBH) Clinical Research Ethics Committee. Written informed consent was waived due to the retrospective nature of this study, and no personal information was disclosed.

## Author contributions

Conception and design: S-QC, Y-QZ, KW, J-KF, and L-YY; administrative support: S-QC; provision of study materials or patients: S-QC and CL; collection and assembly of data: Y-QZ, KW, J-KF, L-YY, Y-JX, XW, and F-FM; data analysis and interpretation: Y-QZ, J-KF, and L-YY; manuscript writing: all authors. All authors contributed to the article and approved the submitted version.
